# On Certain Animal Extracts—Their Mode of Preparation and Physiological and Therapeutical Effects

**Published:** 1893-03

**Authors:** William A. Hammond

**Affiliations:** Washington, D. C.


### *ORIGINAL COMMUNICATIONS.*

### ON CERTAIN ANIMAL EXTRACTS—THEIR MODE OF PREPARATION AND PHYSIOLOGICAL AND THERAPEUTICAL EFFECTS.*

By WILLIAM A. HAMMOND, M. D.,
WASHINGTON, D. C.

I want to tell you of some of the work upon which I have been engaged since I left you, and the story will, I think, interest a body of physicians like yourselves, who come here to learn new facts, and thus to keep abreast with the progress of the age. You remember that about three and a half years ago Dr. Brown-Séquard electrified the medical and non-professional world by announcing that the expressed juice of the testicles of the guinea pig was an agent capable, when injected into the blood, of arresting to some extent the inroads of old age and of curing certain diseases to which mankind is subject. I at once entered upon a series of investigations of the matter, some of the results of which are published in the *New York Medical Journal* for August 13, 1889. I became convinced that we had in the juice of the organs in question a means of acting upon the body in a manner and to an extent different from that of the effects of any other substance previously known to medical science.

But, though surprising in its action, I found that there were certain practical difficulties in the way of the fresh testicular juice ever becoming of general use in actual practice.

In the first place, it had to be used fresh, for if not, there was

*A lecture delivered at the New York Post-Graduate Medical School and Hospital, January 16, 1893, by William A. Hammond, M. D., surgeon-general United States army (retired), late professor of diseases of the mind and nervous system in that institution.

great danger of a putrefactive process being set up and blood poisoning produced, and this was the result in several cases in which it was used in this country. In large cities there is almost an impossibility of getting the organs in question immediately on their being removed from the animal.

Secondly, it was extremely difficult to filter the thick juice, even when diluted according to Brown-Séquard's directions. Filtering paper would not do, for the morphological constituents passed through and an abscess was very liable to be produced at the point of injection. A porous stone filter absorbed the juice, and none of it came through, as there was never a sufficient quantity to saturate the stone and to pass through it. A large amount could not properly be made at one time, as it would not keep, so that it was necessary at every séance to prepare a fresh quantity.

After a time, therefore, during which I did my best with the fresh juice, using for this purpose the testicles of the ram and creating several abscesses with febrile disturbance, I gave up this method and turned my attention to preparing extracts not only of the testicles, but of other organs of the body. It would be to some extent instructive to go over my failures, but I have not time for that. I can only on this occasion tell you of my success and the conclusions I have arrived at in regard to the subject. And I shall mainly confine my remarks at present to the consideration of one extract, that of the brain, which for convenience I designate "cerebrine." I will merely say that I have prepared extracts also of the spinal cord, "medulline" ; the testicles, "testine" ; the ovaries, "ovarine" ; the pancreas, "pancreatine" ; the stomach, "gastrine" ; and the heart, "cardine" ; and that I am nearly ready to give to the profession the results of my observations with these substances. Of course, the kidneys and the liver being excretory organs, cannot properly be used for the purpose of making extracts to be introduced into the blood. Were we to use them in this manner we should be putting back into the system poisons which it had eliminated, and hence would produce disaster, and perhaps even death.

The process and preparation of the extract of these several organs, while individually somewhat different, does not materially vary from that used for the brain, which is as follows :

The whole brain of the ox after being thoroughly washed in water acidulated with boric acid, is cut into small pieces in a mincing machine. To one thousand grammes of this substance placed in a wide-mouthed, glass-stoppered bottle, I add three thousand cubic centimeters of a mixture consisting of one thousand cubic centimeters each of a saturated solution of boric acid in distilled water, pure glycerine and absolute alcohol. This is allowed to stand in a cool place for at least six months, being well shaken or stirred two or three times a day. At the end of this time it is thrown upon a porous stone filter, through which it percolates very slowly, requiring about two weeks for entirely passing through. The residue remaining upon the filter is then enclosed in several layers of aseptic gauze, and subjected to a pressure of over a thousand pounds, the exudate being allowed to fall upon the filter, and mixed with a sufficient quantity of the filtrate to cover it. When it is entirely filtered, it is thoroughly mixed with the first filtrate and the process is complete.

During the whole of this manipulation the most rigid antiseptic precautions are taken. The vessels and instruments required are kept in boiling water for several minutes, and are then washed with a saturated solution of boric acid. Bacteria do not form in this mixture under any circumstances, but it is necessary to examine it from time to time microscopically, in order to see that no foreign bodies have accidentally entered. Occasionally, owing to causes which I have not determined, though I think it is due to variations in temperature, the liquid becomes slightly opalescent from the formation of a flocculent precipitate. It sometimes takes place in a portion of the extract kept under apparently identical conditions with other portions that remain perfectly clear. It can be entirely removed by filtration through Swedish filtering paper, previously sterilized, without the filtrate losing any of its physiological or therapeutical power.

Five minims of this extract diluted at the time of injection with a similar quantity of distilled water constitute a hypodermic dose.

The most notable effects on the human system of a single dose are as follows, though in very strong, robust and large persons a somewhat larger dose is required, never, however, exceeding ten minims :

1. The pulse is increased in the course of from five to ten minutes, or even less in some cases, by about twenty beats in a minute, and is rendered stronger and fuller. At the same time there is a feeling of distention in the head, the perspiration is largely increased, the face is slightly flushed, and occasionally there is a mild frontal, vertical or occipital headache, or all combined, lasting, however, only a few minutes.

2. A feeling of exhilaration is experienced which endures for several hours. During this period the mind is more than usually active and more capable of effort. This condition is so well marked that if a dose be taken about bedtime wakefulness is the result.

3. The quantity of urine excreted is increased, when other things are equal, by from eight to twelve ounces in the twenty-four hours.

4. The expulsive force of the bladder, and the peristaltic action of the intestines are notably augmented, so much so that in elderly persons in whom the bladder does not readily empty itself without considerable abdominal effort, this action is no longer required, the bladder discharging itself fully and strongly, and any existing tendency to constipation disappears, and this to such an extent that fluid operations are often produced from the rapid emptying of the small intestine.

5. A decided increase in the muscular strength and endurance is noticed at once. Thus, I found in my own case that I could "put up" a dumb-bell weighing forty-five pounds fifteen times with the right arm and thirteen times with the left arm, while after a single dose of the extract I could lift the weight forty-five times with the right arm and thirty-seven times with the left arm.

6. In some cases in elderly persons an increase in the power of vision is produced, and the presbyoptic condition disappears for a time.

7. An increase in the appetite and digestive power. Thus, a person suffering from anorexia and nervous dyspepsia is relieved of these symptoms, temporarily, at least, after a single dose hypodermically administered.

These effects are generally observed after one hypodermic injection, and they continue for varying periods, some of them lasting for several days. In order that they may be more enduring, two doses a day should be given every day or every alternate day, as may seem necessary, one in the morning and one in the afternoon, and kept up as long as the case under treatment seems to require. The most notable effects are seen in the general lessening of the phenomena accompanying advancing years. When some special disease is under treatment, the indications for a cessation of the injections will be sufficiently evident, either by an amelioration or cure.

To the substance obtained in this manner and held in solution, I have given, as stated, the name of "cerebrine" as the one, in view of its origin, most appropriate.

I have employed the solution of "cerebrine" with curative effects in many diseases of the brain and nervous system. It is almost specific in those cases of nervous prostration—the so-called neurasthenia—due to reflex causes or excessive mental work, or persistent and powerful emotional disturbance; a hypodermic injection of five minims, twice daily, continued for two or three weeks, and without other medicine, being sufficient to produce a cure. It has proved equally effectual in cases of cerebral congestion, in which the most prominent symptom was insomnia, sleep being produced usually in the course of two or three nights. I have also employed it successfully in migraine, hysteria, melancholia, hebephrenia—the mental derangement occurring in young people of either sex at the age of puberty—in old cases of paralysis, the result of cerebral hemorrhage. In neuralgia, sciatica, and in lumbago, it has acted like a charm, except in one case of facial neuralgia, in which it did not appear to be of the slightest service.

I have employed it in eleven cases of epilepsy. Three of these were of the *petit mal* variety; in two the effect has been so marked that I am not without the hope that cures will result, although I am not able, as yet, to speak positively on this point, the patients having been less than a month under treatment. In the other, no influence appeared to be produced.

Eight cases were of the *grand mal* variety. In two of these the number of paroxysms has been reduced more than one-half, and greatly mitigated in severity. In six other cases which were of long duration I could perceive no curative effects.

In a case of general paresis no therapeutical influence was apparent beyond that of arresting the delusions of grandeur for a few days. In a case of hebephrenia, however, occurring in the person of a young lady eighteen years of age, the effect has been most happy, the symptoms entirely disappearing in a little more than a month's treatment.

In several cases of nervous prostration, the result of long continued emotional disturbance, and in which there were great mental irritability, dyspepsia, physical weakness, loss of appetite and constipation, relief was rapidly afforded. In three other cases in which the most notable symptom was functional cardiac weakness, the effect has been all that could have been desired. In these cases it was employed in conjunction with "cardine," the extract of the heart of the ox, made in the manner already described.

It is not my intention at the present to bring before you all the points of this interesting subject, or to allude further to experiments in the treatment of other diseases, which are not yet concluded. In the near future I shall enter more largely into the consideration of the matter in all its details. I will only add now that I have used with excellent results in cases in which it seemed to be indicated, the extract of the testicles of the bull and also that of the pancreas of the ox, and these investigations also will be given to the profession at an early day. The first named of these "testine," I have found to be of the greatest efficacy in the treatment of sexual impotence when it has been the result of venereal excesses, and in cases of too frequent nocturnal seminal emissions.

It has recently been alleged by some medical authorities, that there is no difference in the physiological or therapeutical action of medicines, whether they be introduced directly into the blood by hypodermic injections or taken into the stomach, but it is scarcely worth while to seriously combat this assertion. For while it may be true that some substances are not altered by the gastric juice before they are absorbed into the system, it certainly is not true of many others, and it surely is erroneous as regards those of animal origin. Indeed it is, I think, doubtful if anything capable of being acted upon by the gastric juice and of being absorbed into the blood gets into the system in exactly the same form in which it got into the stomach. And I am very sure that all organic matters, without exception, undergo radical changes under the action of the gastric juice, in some cases amounting to decomposition and recomposition.

It is well known that Woorara, the virulent arrow poison used by the Indians of South America, and which is invariably fatal to animal life when injected into the blood, is innocuous when taken into the stomach, even in very large quantity. I have ascertained by actual experiment that the poison of the rattlesnake may be swallowed with impunity. During the course of my medical service in the army on the western plains, I have collected a large quantity of rattlesnake poison. A small fraction of a grain of this injected hypodermically was sufficient to kill a dog in a few minutes, while previously the same animal had been made to swallow a half a drachm without the production of any apparent result. Experiments made with the saliva of hydrophobic animals prove that it is rendered harmless by the action of the gastric juice. The vaccine virus may certainly be swallowed with impunity, as has been shown by repeated experiments upon animals.

Relative to the animal extracts to which I am now referring, I have ascertained beyond question that if they are inclosed in capsules so as to reach the stomach without coming in contact with the mucous membrane of the mouth, they are absolutely without physiological or therapeutical effect so far as can be perceived, even when given in quantities of a teaspoonful or more, but if dropped upon the tongue in double the quantity used for hypodermic injection and allowed to remain in the mouth without being swallowed, thus avoiding the action of the gastric juice, they are absorbed and exert a slower but still decided effect, though nothing comparable to that produced when they are administered hypodermically.

Now, gentlemen, a few words in regard to the theory upon which these animal extracts exert these remarkable effects. I have thought a good deal upon the matter and I think I have arrived at something like the truth. But after all a theory, even when supported by indisputable facts, is not a matter of so much importance as the facts themselves. And it is better if you are sure of your facts to have an erroneous theory than none at all. The one I am going to propose is, I think, in accordance with physiological law, and I believe that it will strike your minds as being based on common sense, and as being sufficient to account for the observed phenomena. Briefly stated, it is as follows:

Organic beings possess the power of assimilating from the nutritious matters they absorb the peculiar pabulum which each organ of the body demands for its development and sustenance. The brain, for instance, selects that part which it requires; the heart the material necessary for its growth and preservation, and so on with the liver, the lungs, the muscles and the various other organs of the body. No mistake is ever committed; the brain never takes liver nutriment nor the liver brain nutriment, but each selects that which it requires. There are, however, diseased conditions of the various organs in which this power is lost or impaired, and as a consequence disturbance of function, or even death itself, is the result.

Now, if we can obtain the peculiar matter that an organ of the body requires and inject it directly into the blood, we do away with the performance of many vital processes which are accomplished only by the expenditure of a large amount of vital force.

Let us suppose a person suffering from an exhausted brain, the result of excessive brain work. Three hearty meals are eaten every day, but no matter how judiciously the food may be arranged the condition continues. Now, if we inject into that person's blood a concentrated extract of the brain of a healthy animal, we supply at once the pabulum which the organ requires. Then, if under this treatment the morbid symptoms disappear, we are justified in concluding that we have successfully aided nature in doing that which unassisted she could not accomplish.

All this is applicable not only to the brain, but certainly to the heart, the generative system, the spinal cord and, I believe, other organs of the body. I have repeatedly seen a feeble heart rendered strong, the blood corpuscles increased in number and the color of the blood deepened by the use of cardine, and I have many times seen an exhausted sexual system restored to its normal power by the use of testine, cerebrine and medulline.

Such is the system, and yet I am not quite sure that it is entirely new. I recollect reading nearly forty years ago an account of some observations made by, I think, a German physician relative to the treatment of diseases of the several organs of the body by a system of diet consisting of the corresponding organs of healthy animals. Thus liver disease was treated by beef's liver, heart disease by beef's heart, brain disease by beef's brain and so on. My memory seems to be clear on the main point, but I have searched in vain for the paper to which I refer. The fact, however, that the various foods in question were cooked and were taken into the stomach constitutes a great difference with the system which I am now discussing, both physiologically and therapeutically, and the results do not admit of comparison. The germ of the idea, however, is the same, and I cheerfully yield to my unknown proto-observer whatever distinction may be claimed on the score of priority.

And while I have been conducting my observations others have been at work in the same direction, but their investigations do not seem to have led to any very definite results or to have been systematically carried out. Generally they have been performed with the fresh juice of the organs, and although at first sight this method would appear to be preferable to any other, experience shows that it is, as I have said, not unattended with danger, and I have certainly ascertained that extracts made with glycerine and pressure, extemporaneously, are absolutely without effect, either physiologically or therapeutically.

